# The emerging role of galectins in (re)myelination and its potential for developing new approaches to treat multiple sclerosis

**DOI:** 10.1007/s00018-019-03327-7

**Published:** 2019-10-18

**Authors:** Charlotte G. H. M. de Jong, Hans-Joachim Gabius, Wia Baron

**Affiliations:** 1 Department of Biomedical Sciences of Cells & Systems, Section Molecular Neurobiology, University of Groningen, University Medical Center Groningen, A. Deusinglaan 1, 9713 AV Groningen, The Netherlands; 2 Institute of Physiological Chemistry, Faculty of Veterinary Medicine, Ludwig-Maximilians-University Munich, Munich, Germany

**Keywords:** Galectins, Multiple sclerosis, Myelination, Oligodendrocytes, Remyelination

## Abstract

Multiple sclerosis (MS) is an inflammatory, demyelinating and neurodegenerative disease of the central nervous system with unknown etiology. Currently approved disease-modifying treatment modalities are immunomodulatory or immunosuppressive. While the applied drugs reduce the frequency and severity of the attacks, their efficacy to regenerate myelin membranes and to halt disease progression is limited. To achieve such therapeutic aims, understanding biological mechanisms of remyelination and identifying factors that interfere with remyelination in MS can give respective directions. Such a perspective is given by the emerging functional profile of galectins. They form a family of tissue lectins, which are potent effectors in processes as diverse as adhesion, apoptosis, immune mediator release or migration. This review focuses on endogenous and exogenous roles of galectins in glial cells such as oligodendrocytes, astrocytes and microglia in the context of de- and (re)myelination and its dysregulation in MS. Evidence is arising for a cooperation among family members so that timed expression and/or secretion of galectins-1, -3 and -4 result in modifying developmental myelination, (neuro)inflammatory processes, de- and remyelination. Dissecting the mechanisms that underlie the distinct activities of galectins and identifying galectins as target or tool to modulate remyelination have the potential to contribute to the development of novel therapeutic strategies for MS.

## Introduction

Multiple sclerosis (MS) is a heterogeneous inflammatory, demyelinating and neurodegenerative disease of the central nervous system (CNS) that affects 2.5 million people worldwide. The most common clinical form is relapsing-remitting MS (RR-MS, 85%). Patients endure phases of increasing neurological deficits followed by recovery periods. After some time, approximately 60% of the patients enter a phase that is characterized by a steady decline of neurological functions with or without relapse (secondary progressive MS, SP-MS). Neuronal loss and disease progression are irreversible at this phase. Primary progressive MS (PP-MS) affects a subset of patients (10–15%) that is characterized by continuous progression of the disease from its onset. Current treatments are disease-modifying therapies and encompass application of immunosuppressive or immunomodulating drugs that reduce the number and severity of relapses in RR-MS, but these interventions are ineffective in halting disease progression [1–3]. Hence, there is an obvious need to develop new therapeutic strategies for progressive MS.

Remyelination following demyelination is essential for axonal survival and restoration of saltatory conduction [4–8], and its failure is a major cause of the neurological deficits in MS [9–12]. Therefore, restoring remyelination could prove to be an effective treatment in reversing disability and halting disease progression. Toward the aim of designing effective therapies that induce remyelination, it is important to understand the biological mechanisms that underlie the remyelination process and to identify factors that prevent remyelination in MS. Remyelination fails despite the presence of oligodendrocyte progenitor cells (OPCs) in most lesions [13–19]. This observation implies that either stimulating extrinsic or intrinsic factors are absent or that inhibitory signals are dominant [20–23]. In principle, this reasoning prompts to examine receptor-driven pathways and routes of communication between cells.

In terms of a recognition process of broad relevance, the abundance of glycoconjugates in the nervous system directs attention to considering the glycan part of glycolipids and glycoproteins as versatile ligand (for introduction into these glycan structures, please see [24–31]). In fact, the concept of the sugar code assigns an unsurpassed ability to store information to these glycans. Tissue receptors (lectins) are present that will ‘read’ these sugar-encoded signals, followed by ‘translation’ into effects, eliciting a broad variety of post-binding activities [32–34]. This functional pairing does not only depend on the complementarity of the direct ligand(glycan)–receptor(lectin) contact but also on topological parameters to achieve the inherently high levels of selectivity and specificity, letting only certain glycoconjugates with distinct (cognate) glycan display become counterreceptors for a tissue lectin [35, 36]. Following this reasoning, that is, a function of this interplay in “establishment of the cell–cell contacts and possibly also as mediators of communication between the surface and the interior of the cell”, and the abundance of glycoconjugates in the nervous system, extracts of the electric organ tissue of *Electrophorus electricus* proved to be the source of a lectin specific for β-galactosides that became the first member of the ga(lactose-binding)lectin family [37].

These galectins are special to exert activities inside and outside of cells by glycan- and via protein-dependent binding so that they are multifunctional [38–45]. Targeting their counterreceptors, forming molecular bridges between them in adhesion (between cells) or lattice establishment (on the membranes’ surface) and hereby triggering signaling fulfills criteria for being a versatile effector. Proceeding from work on individual galectins to a network analysis is teaching the lesson that they can be expressed at the same sites and can functionally cooperate [46, 47]. Thus, their study is a step to give meaning to the expression of certain glycans at distinct sites and to aberrations of the glycome related to the disease [48]. With focus on (re)myelination and the (immuno)pathophysiology of MS, galectins have already attained the status of notable players in this context. This review first provides an introduction to this class of effectors and then describes known roles of galectins during developmental myelination, remyelination and in the course of MS. In this context, the current status of knowledge on what galectins do, particularly in modulating immune responses and behavior of CNS glial cells, i.e., oligodendrocytes, astrocytes and microglia that are relevant to (re)myelination, is summarized as well as the relevance of galectins for MS pathology. Finally, we discuss how galectins, either as targets or tools, may help to inspire the development of novel therapeutic strategies to combat remyelination failure in MS and hence to halt disease progression.

## Introduction to galectins

Galectins are a family of evolutionarily conserved proteins that share β-sandwich folding and a distinct sequence signature within the carbohydrate recognition domain (CRD). Beyond binding the canonical ligand lactose/*N*-acetyllactosamine (Lac/LacNAc), phylogenetic diversification has led to a divergence of the carbohydrate-binding profiles, for example, studied using frontal affinity chromatography or glycan arrays [49–52]. In principle, glycans of glycoproteins such as suited *N*-glycan, mucin-type *O*-glycan or *O*-mannosylated chains or of glycolipids serve as contact partners. Introduction of substituents such as a sulfate group or a sialic acid can serve as a switch for ligand activity. Of note, dynamic enzymatic interconversions from a cryptic to an active site for docking, for example, by desialylation [53], or spatiotemporally regulated shifts in the glycome ensure flexibility in controlling the recognition potential swiftly. Teaming up with the ligand specificity of the galectins, protein architecture is relevant for the nature of triggered post-binding activities, as recently highlighted by the design of custom-made variants of human galectins [54, 55]. Thus, it is important to learn about galectins’ properties in this respect.

**Fig. 1:**
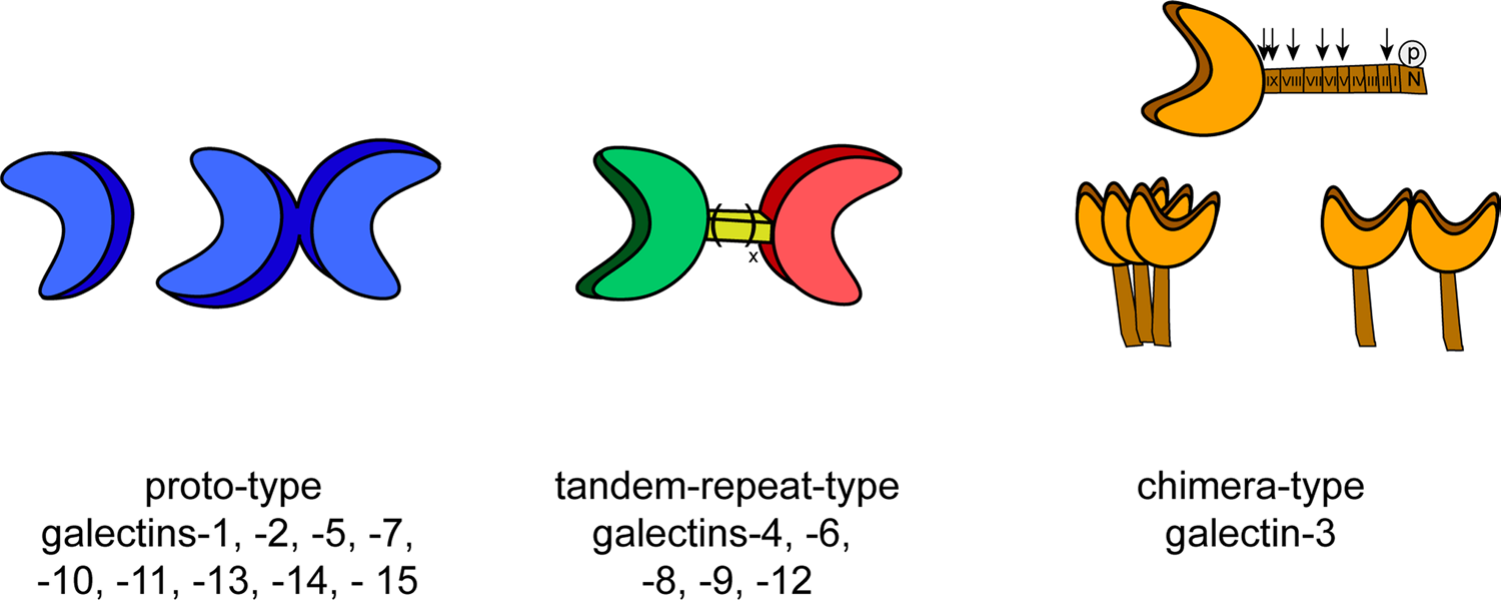
Overview of the classification of the three types of modular architecture of vertebrate galectins. Proto-type galectins contain a single carbohydrate recognition domain (CRD) and are able to form monomeric or homodimeric structures. Tandem-repeat-type galectins have two distinct CRDs and are covalently associated via a linker peptide with natural variation of linker length by alternative splicing; chimera-type galectin, i.e., galectin-3 harbors one CRD and a non-lectin domain which consists of an N-terminal region and nine collagen-like repeat units that are substrates for matrix metalloproteinases (MMP-2/-7/-9/-13) and PSA-mediated cleavage at different positions shown by arrows. The N-terminal region functions as a site for serine phosphorylation. Galectin-3 is monomeric in solution in the absence of a ligand and can form aggregates in contact to oligo- or polyvalent ligands via the N-terminal tail, the CRD, or both

As illustrated in Fig. 1, three types of protein structures form the set of galectins in vertebrates. Notably, non-covalently associated homodimers, linker-connected heterodimers and a structural chimera of a CRD with an N-terminal tail (consisting of non-triple-helical collagen-like repeats enabling self-interaction and a sequence bearing two sites for serine phosphorylation) facilitate to bring together ligands in different constellations and topological order [56]. The chimera-type galectin-3 (Gal-3) is thus special to build aggregates of different spatial order via contacts between CRDs or the tail, which is a substrate for various proteases that shorten its length and impair aggregation [57–61]. In summary, galectins combine target specificity with the ability to generate molecular associations at various sites of the cell.

As first described for galectin-1 [62], galectins are synthesized in the cytosol, then reaching destinations such as the nucleus, diverse binding partners in the cytoplasm or glycans on damaged vesicle surfaces [63–66]. Overall, family members such as galectins-1 and -3 can thus perform multiple activities that depend on their cellular localization regulating cell cycle, survival (via binding of bcl-2) and RNA processing [67–69]. In addition, despite commonly lacking a secretion signal peptide, galectins are secreted into the extracellular space and this by non-classical pathways [70, 71] that, for example, involve exosomes [72–74]. Once secreted, galectins bind to matrix or cell surface glycoconjugates, readily bridging suited partners to form aggregates, and this is regulated by glycan structure, density and mode of presentation [75, 76]. When then in contact with the cell surface, galectins can re-enter the cell, there handled by the trafficking machinery as elaborately as for export and involved in sorting basolateral and apical cargo in post-Golgi compartments [77, 78]. Hereby, the residence time of counterreceptors on the surface is intimately regulated, in critical dependence of the presence of cognate glycans. Underscoring the physiological potential of galectins, their presence is under strict control, and first cases have been described for an intimate spatiotemporal co-regulation of galectin/counterreceptor presentation, for example, the Gal-1/ganglioside GM1 route of communication between effector/regulatory T cells and in axon growth induction [79, 80]. This survey explains why it is likely that galectins will also be important in CNS processes.

Since galectins are also very potent regulators of (neuro)inflammation, a dysregulation of galectins is expected to be associated with several neuroinflammatory diseases. Thus, examining the hypothesis of galectins as potent regulators of developmental myelination and remyelination as well as of a role in MS pathology is of relevance.

## Role of galectins in developmental CNS myelination

### Regulation of developmental myelination: a major role for neurons

Oligodendrocytes are the myelinating cells of the CNS and essential for saltatory conduction and axon survival [4–8]. They are generated from OPCs, which arise from neural stem cells in the subventricular zone [81]. Via the influence of a complex network of attractants and repellents such as semaphorins, OPCs proliferate and migrate via three consecutive waves throughout the developing CNS [82]. In addition, OPCs need the physical interaction with the vascular endothelium to migrate to their destination [83]. When having arrived at their destination and then subjected to local, mainly neuron-derived signals, OPCs start to differentiate towards mature, post-mitotic myelinating oligodendrocytes. Notably, part of the OPCs persist in the adult brain and develop into adult OPCs, while the generation of OPCs from neural stem cells also continues into adulthood [81, 84, 85]. The differentiation phase consists of (1) establishing contact with the newly formed axon, (2) expressing myelin genes and generating myelin membranes and (3) enwrapping the axon and creating a compacted myelin sheath. OPC differentiation requires appropriate timing for its initiation and then follows stepwise stages. The course of differentiation is well studied and defined in cultured OPCs by morphology and by the appearance of stage-specific lipid and protein markers [86–88]. Viewing such characteristics, OPCs are bipolar and distinguished from mature oligodendrocytes by the expression of platelet-derived growth factor receptor alpha (PDGFRα), neural/glial antigen 2 (NG2), surface gangliosides that are recognized by A2B5 antibody, and transcription factor NK2 Homeobox 2 (Nkx2.2). Concerning this aspect of the proteome, oligodendrocyte lineage cells share the expression of the oligodendrocyte transcription factor 2 (Olig2) [89, 90]. Immature oligodendrocytes are in an intermediate status of differentiation, and here the expression of 2′3′-cyclic nucleotide 3′-phosphodiesterase (CNP), a myelin-specific protein, and glycosphingolipids serve as characteristics. At this stage, the cells present high levels of galactosylceramide (GalCer) and its derivative with 3′-*O*-sulfation, i.e., sulfatide, at their surface. However, they do not form myelin membranes yet. Mature oligodendrocytes then have multiple processes and generate myelin membranes, while maintaining high levels of sulfatide and GalCer at their surface. On the level of proteins, mature oligodendrocytes are characterized by the expression of myelin constituents, including the major myelin basic protein (MBP) and proteolipid protein (PLP) that are present in compact myelin and myelin-associated glycoprotein (MAG) and myelin oligodendrocyte glycoprotein (MOG) that are present in non-compact myelin.

Both extrinsic factors and intrinsic signaling mechanisms that can engage transcription factors control OPC differentiation. The onset of OPC differentiation at the appropriate time and place is explained by the “derepression” model [91]. Central to it, transcription factors that maintain the status are downregulated or they are relocalized by reducing extrinsic signals that constantly inhibit differentiation. This prevents premature OPC differentiation and allows for a tightly regulated timing of OPC differentiation by stimulating factors. During development, inhibitory factors for OPC differentiation are mainly axon derived. In fact, there are several means by which axons affect OPC behavior and the correct onset of OPC differentiation. For example, inhibitory axonal factors inhibit premature OPC differentiation such as Jagged-1, neural cell adhesion molecule-bearing polysialic acid (α2,8-linked sialic acids; PSA) chains [92, 93] and LINGO-1 (leucine-rich repeat Ig domain-containing Nogo-interacting protein 1) [22, 94, 95]. Besides the axonal inhibitory signals that determine differentiation onset, axons secrete trophic factors [such as PDGF, fibroblast growth factor 2 (FGF-2), insulin-like growth factor 1 (IGF-1)] that regulate OPC proliferation and migration [96–98]. Myelin formation and OPC differentiation are promoted by the release of glutamate from synaptic vesicles along axons in vitro [99–101]. It appears that synapses onto myelin-forming oligodendrocytes are not required for activity-dependent myelination. In contrast, myelination is regulated by non-synaptic junctions that signal through local intracellular calcium [102]. Glutamate release from active axons initiates local production of MBP in oligodendrocytes by the assembly of cholesterol-rich microdomains and induction of Fyn kinase activity [99]. In addition, an increase of frequency of Ca^2+^ transient activity in sheaths is correlated with sheath elongation [103]. Interestingly, a certain oligodendrocyte is able to compartmentalize signals as different processes of the cell that act independently regarding myelin induction [102]. However, multiple stages are involved in the formation of myelin and only within a brief window of opportunity will oligodendrocytes generate new myelin segments [104, 105].

Next to neuronal-derived signals, communication of astrocytes and microglia to oligodendrocytes contributes to developmental myelination and myelin maintenance [106–108], while being even more prominently involved in the regulation of remyelination (see in section “Role of galectins in CNS remyelination”). Also, adaptive immune cells are involved in developmental myelination. B cells migrate to the developing brain and increase OPC proliferation by the secretion of natural IgM antibodies [109]. While the molecular and cellular regulation of developmental myelination has been studied extensively, insights into the role of the glycome and of galectins in neuronal function and OPC maturation herein are being gained over a comparatively brief period. Major steps toward defining galectins as parts of the machinery driving these processes are presented in Table 1.

**Table 1:** Galectins during developmental myelination and upon de-and remyelination

Galectin	Model	Main result	Mechanism	References
In vivo				
gal-1	*Lgals1*^−*/*−^ mice (C57Bl/6)	Less and more loosely wrapped myelinated axons	Controls myelin compaction and integrity	[156]
gal-1	Lysolecithin-induced demyelination (C57Bl/6 mice, treatment)	Reduced demyelination and improved remyelination	Shifts microglia towards a regenerative phenotype, increases phagocytosis of myelin debris and OPC differentiation	[156]
gal-3	*Lgals3*^−*/*−^ mice (C57Bl/6)	Decreased percentage myelinated axons, myelin turns and g-ratio. Loosely wrapped and less smooth myelin	Required for proper production and organization of myelin	[123]
	*Lgals3*^−*/*−^ mice (129 Sv)	No effect on OPC differentiation upon development		[220]
gal-3	Cuprizone-induced demyelination (*Lgals3*^−*/*−^ C57BL/6 mice)	Decreased OPC differentiation, enhanced reactive astrogliosis, defective microglia activation and hypomyelination	Inability to upregulate the phagocytic receptor TREM-2b on microglia and decreased MMP-3 expression	[151, 221]
	Cuprizone-induced demyelination (*Lgals3*^−*/*−^ 129Sv mice)			
		Increased emigration of SVZ cells to demyelinated areas and no effect on OPC differentiation	Controls local inflammation in the SVZ and limits SVZ progenitor emigration	[220]
gal-4	Cuprizone-induced demyelination (C57Bl/6 mice)	Re-expressed in axons and present in microglia/macrophages	Neuronal re-expression and secretion of gal-4 may inhibit OPC differentiation	[124, 179]
In vitro				
gal-1	Astrocytes (primary cell culture F344/N Slc rats, treatment)	Induces differentiation and inhibits proliferation	Increases production of BDNF	[217]
gal-1	Oligodendrocytes (primary cell culture, Wistar rats, treatment)	Low concentrations inhibit OPC differentiation	Upregulates MMP-2 activity in conditioned medium of immature oligodendrocytes that may cleave gal-3′s N-terminal tail	[123, 167]
		High concentrations enhance OPC differentiation	May increase OPC viability upon cell cycle exit	
gal-3	Oligodendrocytes (primary cell culture, Wistar rats, treatment)	Promotes OPC differentiation	Gal-3′s N-terminal tail is cleaved by MMP-2 in OPCs, but not mature oligodendrocytes, gal-3 induces actin filament assembly and drives early branching of oligodendrocyte processes	[123, 167]
gal-3	Microglia (*Lgals3*^*−/*−^ C57BL/6 mice)	Microglia-conditioned medium with secreted gal-3 promotes OPC differentiation	Microglia-expressed gal-3 favors an anti-inflammatory phenotype	[123, 158]
gal-4	Oligodendrocytes (primary cell culture, Wistar rats, treatment)	Inhibits OPC differentiation	Direct binding of gal-4 to the OPC (protein integrity with both CRDs and linker is required)	[124]
	Oligodendrocytes (CG4 cells, primary cell culture)	Enhances MBP promotor activity	Involved in p27- and Sp1-mediated activation of MBP	[148]
gal-4	Cortical neurons (primary cell culture, co-culture with oligodendrocytes, Wistar rats)	Required for proper axon growth and elongation	Sorts and organizes transport of axonal L1 in a sulfatide-dependent manner	[125]
		Gal-4 deposits on axons inhibit myelination	Possible role in recruitment of contactin-1 and correct targeting of nodes of Ranvier	[134]

*BDNF* brain-derived neurotrophic factor, *gal* galectin, *MMP* matrix metalloproteinase, *OPC* oligodendrocyte progenitor cell, *SVZ* subventricular zone

### Galectins in neuronal function

Initial evidence for galectin presence in neurons by haemagglutination assays [110–112] led to immunohistochemical localization [113, 114] and application of a galectin as tool for detecting accessible binding sites [115]. Intriguingly, lactoseries glycoconjugates appear available so that a functional pairing was hypothesized within the concept of the sugar code already at that time [116]. In this context, maturation of neurons during CNS development involves directed axonal growth towards the correct targets, accompanied by neurite branching necessary for an exploration of the environment. At present, galectins-1, -3 and -4 have been shown to be instrumental in axonal development and functioning including its myelination. Galectin-1 is prominently expressed in neurons and upregulated during sensory and motor neuron development [117, 118]. Its presence guides primary olfactory and somatosensory axons and promotes neurite sprouting, both in vitro and in vivo, i.e., as shown by aberrant topography of olfactory axons in *Lgal1*^−*/*−^ mice [117, 119–121]. Galectins-3 and -4 are transiently expressed during development and downregulated at the onset of myelination [122–124]. Galectin-4 is present in cortical and olfactory neurons [124], here required for proper axon growth and elongation [125]. In functional terms, neuronal galectin-4 sorts and organizes transport of the axonal glycoprotein neural cell adhesion molecule L1 in a sulfatide-dependent manner [125]. Galectin-4, via binding to LacNAc termini of *N*-glycans, ensures proper clustering of L1 on axons in membrane microdomains and spatial organization at the axonal surface [125]. As observed in polarized epithelial cells, neuronal galectin-4 stabilizes distinct membrane microdomains and organizes apical protein transport of its cargo L1 [77, 126]. Of note, in cultured hippocampal neurons, L1 binds to immobilized galectin-3 when phosphorylated at the serine residues in the N-terminal section, what in turn regulates the segregation of L1 to discrete plasma membrane domains [127]. These domains recruit membrane–actin linkers (ERMs), which destabilize actin to stimulate local axon branching. In addition, extracellular immobilized galectin-3 promotes neurite outgrowth, but—in contrast to galectin-1—has no effect on axonal guidance in vitro [127–129]. When appropriately clustered, L1 binds to oligodendroglial contactin (also called F3) and activates Fyn kinase, which initiates MBP-specific mRNA synthesis and myelin biogenesis in oligodendrocytes [130–133]. In addition, axons harbor discrete galectin-4-containing domains that impede the deposition of myelin by oligodendrocytes [134]. In these myelination-excluding domains, galectin-4 interacts with axonal contactin-1, which in myelinated axons is present in the non-myelinated nodes of Ranvier [134]. Interestingly, the sequestering of the nodal protein contactin-1, the expression of neuronal galectin-4, and the size of the galectin-4-containing domains are independent of the interaction with oligodendrocytes or myelin, indicating that this is an intrinsic property of neurons. Hence, endogenous galectin-4 modulates axonal formation and outgrowth and it precludes myelin deposition, while exogenous galectins-1 and -3 determine the extent and position of axon branching. Obviously, these data do not only indicate physiological significance of individual galectins, but also substantiate functional cooperation so that further exploring the galectin network, for example, following initial data on RT-PCR signals for galectins-7 and -8 [135], is an attractive endeavor.

### Galectins in oligodendrocyte maturation

In addition to the role of endogenous neuronal galectin-4 as a local axonal inhibitor of myelination, secreted neuronal galectin-4 regulates the timing of OPC differentiation and therefore the onset of myelination. Non-myelinated neurons produce and secrete galectin-4, which then binds to still uncharacterized counterreceptors that transiently appear on primary processes of immature oligodendrocytes [124]. Extracellular galectin-4 binding impairs OPC differentiation and induces dedifferentiation and proliferation in a subset of cells. Both CRDs of the heterodimeric galectin that are associated by a linker of a length of physiological significance [136], and the integrity of this display as tandem-repeat-type protein are required for galectin-4-mediated inhibition of OPC differentiation [124]. This result suggests that galectin-4 may reorganize the membrane by bringing distinct glycoconjugates in close proximity exclusively at the cell surface of primary processes. Given its association with axonal contactin-1 [134] and that oligodendroglial F3/contactin-1 triggers MBP expression [130, 131, 133, 137], it is tempting to assume that one of the galectin-4-binding sites on the oligodendroglia surface may be contactin-1. At the onset of myelination, neurons cease to secrete galectin-4, which creates a permissive environment for OPC maturation and oligodendrocytes to myelinate the bare axons. What triggers the neuron to discontinue secretion of galectin-4 remains to be determined. In other cells, this process is regulated by Src family kinase-mediated phosphorylation of its C terminus [138]. Of relevance in this respect is that no myelin deficits were observed in Src^−/−^, Yes^−/−^ or Lyn^−/−^ mice at postnatal day 28 [139]. This may be due to compensatory mechanisms, or, because earlier time points were not analyzed, potentially accelerated myelination is not revealed yet. However, Src family tyrosine kinase Fyn expression in neurons and oligodendrocytes is important for myelination [140, 141], although Fyn does not appear to be involved in the timing of OPC differentiation [139]. Also, Src kinase activity is upregulated in Fyn^−/−^ mice [142], and it is tempting to explore the role of neuronal Fyn/Src kinases in galectin-4 phosphorylation in relation to its externalization.

Oligodendrocytes endogenously express, but do not secrete, galectin-4 in vitro. In OPCs, galectin-4 is localized to the cytoplasm, and, as OPCs are polarized cells [132, 143], galectin-4 may affect trafficking of apically located glycoproteins and -lipids, as observed in enterocyte-like cells and neurons [77, 125]. This can very well include sulfatide, especially the fraction-bearing long-chain fatty acids. This galactosphingolipid, that is enriched at the oligodendroglial surface, acts as a negative regulator of myelination [144, 145], as galectin-4 does, and it is also involved in the timed trafficking of the major myelin protein PLP to the myelin membrane [146, 147]. Upon OPC differentiation, galectin-4 shifts from a cytoplasmic to a nuclear localization [124]. In the nucleus, galectin-4 regulates the expression of MBP by binding to the transcription factor Sp1 to activate p27-mediated MBP expression [148, 149]. Hence, while neuronal galectin-4 after secretion precludes OPC differentiation, oligodendroglial galectin-4 in nuclei promotes MBP expression. These observations underscore that the location of galectins matters conspicuously.

In addition to galectin-4, galectins-1 and -3 also modulate the maturation of oligodendrocytes. Galectin-3 expression, similar to that of galectin-4, decreases upon developmental myelination, the galectin-1 level instead increases upon brain development and is leveling off in the adult rat brain [123, 150], our unpublished observations). In contrast, in vitro, galectin-1 is downregulated, whereas galectin-3 is upregulated upon OPC differentiation [123]. In addition, cultured astrocytes and microglia harbor galectins-1 and -3. Although monocultures were examined, in situ hybridization studies that confirm endogenous galectin-specific mRNA levels in glial cells in vitro and in vivo are still lacking, as well as proof that these galectins are externalized by cells of the oligodendrocyte lineage will be welcome. In *Lgals3*^−/−^ mice, MBP expression is downregulated, less axons are myelinated and myelin is less compact than in wild-type mice. The hypomyelination phenotype goes along with increased number of OPCs [151] and appears to be reflected by behavioral abnormalities in *Lgals3*^−/−^ mice [123]. Hence, galectin-3 plays a critical role in OPC differentiation, myelin integrity and function, likely via distinct biological processes. For example, in OPCs but not in mature oligodendrocytes, the N-terminal tail of galectin-3 is cleaved by matrix metalloproteinasae 2 (MMP-2) [123], a process shown in Fig. 1. This indicates that different biological functions of endogenous galectin-3 in OPCs and mature oligodendrocytes appear likely. As already noted, MMP-dependent cleavage impairs the N-terminal tail’s capacity toward self-aggregation [152]. In addition, processing may also affect secretion: a MMP-resistant galectin-3 variant was found to be less secreted [70]. Of interest, glycoprotein cross-linking of galectin-3 is required for apical sorting of non-raft-associated proteins, whereas in galectin-3-depleted cells cargo is mistargeted to the basolateral membrane [78]. Of relevance in this respect is that the growing myelin membrane is served by a basolateral trafficking pathway [153–155]. Therefore, galectin-3 may participate in establishing oligodendrocyte polarity, its absence interfering with myelin biogenesis and compaction, as is observed in *Lgals3*^−/−^ mice [123]. Similar to *Lgal3*^−/−^ mice, *Lgals1*^−/−^ mice have significantly less myelinated axons, particularly in smaller diameter axons, while myelin was more loosely wrapped around axons than in wild-type mice [156]. Galectins-1 and -3 do not compensate for each other, indicating that these galectins control myelin integrity and compaction via distinct mechanisms. A case of functional antagonism between them is the inhibition of galectin-1-dependent neuroblastoma growth regulation by galectin-3 [157].

In addition to their endogenous roles, when administered to cell cultures, galectins-1 and -3 interfere with OPC maturation. Exposure in vitro to a relatively low concentration of recombinant galectin-1 impairs OPC differentiation, whereas galectin-3 treatment at the equivalent concentration increases both OPC differentiation and the extent of myelin membrane formation [123, 158]. In contrast, a relatively high concentration of galectin-1, ensuring its presence as homodimer [159, 160], increases OPC differentiation [156]. Similar contrasting effects of different galectin-1 forms have been observed in peripheral nerve injury. Thus, the common homodimeric form of galectin-1 enhances degeneration of neuronal processes in a lectin-dependent manner, whereas oxidized monomeric galectin-1 that lost its capacity to bind sugar promotes axonal regeneration [161, 162]. The distinct biological functions of galectin-1 on OPC differentiation may depend, in addition to its concentration and timing, on the status of its six cysteine residue. When oxidized at these sites, galectin-1 loses its carbohydrate-binding activity [163–166]. Notably, when immature oligodendrocytes are treated with galectin-1 at a relatively low concentration, MMP activity is enhanced which may increase the extent of MMP-mediated cleavage of galectin-3, indicating the possibility for interplay between these galectins in OPC differentiation [123, 158]. Extracellular galectin-3 accelerates OPC differentiation by modulating signaling pathways that lead to changes in actin cytoskeleton dynamics [158]. More specifically, in a CRD-dependent manner, galectin-3 reduces activation of Erk1/2 and increases Akt-mediated β-catenin signaling, an inducer of a shift from polymerized to depolymerized actin. This change in the status of the actin cytoskeleton dynamics is known to drive oligodendrocyte process outgrowth and branching, what is essential to initiate myelin membrane formation [167, 168]. In addition, extracellular galectin-3 increases MBP expression, a process only partially dependent on its CRD, which emphasizes that galectin-3 modulates OPC differentiation via multiple means including protein–protein interactions via its non-lectin part within the chimeric structure [167].

Microglia and astrocytes are cellular sources of secreted galectin-3 [123]. At least in vitro, cells of the oligodendrocyte lineage do not secrete galectin-3 (our unpublished observations). The action of extracellular galectin-3 on oligodendrocytes thus appears to be paracrine rather than autocrine. During development, galectin-3 is transiently present in microglia, and conditioned medium of galectin-3-deficient microglia does not promote OPC differentiation [123]. Oligodendroglial counterreceptors for galectin-3 remain to be identified, but galectin-3-binding sites are known to be present on cell body and processes of bipolar OPCs, with increasing morphology restricted to the cell body [158]. Of relevance, when microglia galectin-3 binds to IGF receptor 1 [169], a receptor that when activated on OPCs promotes differentiation [170–172]. Therefore, galectin-3 may delay its endocytic uptake by cross-linking the IGF receptor on the oligodendroglial cell surface, thereby potentiating IGF receptor signaling that results in enhanced OPC differentiation.

Taken together, both endogenous galectins and galectin–glycan interactions at the cell surface drive oligodendrocyte maturation. Strikingly, extracellular galectins-1, -3 and -4 modulate OPC differentiation, rationalizing their potential as novel therapeutic targets and/or tools to modulate OPC differentiation in disease. However, as galectins-3 and -4 are transiently expressed during development, their roles upon CNS demyelination and successful remyelination need to be resolved to verify and/or understand the role of galectins in MS pathology (Table 1).

## Role of galectins in CNS remyelination

### Regulation of remyelination: a major role of microglia and astrocytes

Demyelination is the degeneration of myelin sheaths which in the healthy CNS is followed by a spontaneous regenerative response, called remyelination. This process covers the regeneration of complete, newly formed myelin sheaths that enwrap demyelinated axons to reestablish saltatory conduction, which is salient to resolve functional deficits and to prevent axonal degeneration [10, 173–175]. In rodents, remyelination requires the generation of new mature oligodendrocytes from OPCs [176]. Therefore, remyelination morphologically resembles developmental myelination. In fact, some axonal factors including NCAM with its polysialic acid chains (PSA-NCAM), galectin-4 and LINGO-1 that are involved in the regulation of developmental myelination are re-expressed upon injury [20, 22, 177–179]. An additional level of regulatory factors, mainly provided by microglia and astrocytes, is required to limit inflammation and demyelination and to clear myelin debris. More recent studies also point to a direct role of the systemic environment in efficient remyelination, i.e., both circulating TGFβ and regulatory T cells promote OPC differentiation [180, 181]. This distinct regulation of developmental myelination and remyelination is reflected in the formation of shorter and thinner myelin sheaths on remyelinated axons compared to axons that are myelinated upon development.

For successful remyelination to occur, several tightly regulated, well-timed, and distinct sequential steps as well as interplay between distinct types of glial cells and neurons are required. To study the cells and molecular factors involved in remyelination, animal models with global or focally induced demyelination have provided valuable information. Examples of these toxin-induced demyelination animal models are the cuprizone model, where regional demyelination is most prominent in the corpus callosum upon feeding cuprizone [182–185], and the focal lysolecithin model. Here, demyelination is induced by a local injection of lysolecithin in the brain or spinal cord white matter [184, 186, 187]. Importantly, lysolecithin acts also on pericytes which leads to disruption of the blood–brain barrier (BBB) [188], while in the cuprizone model the BBB remains seemingly intact with hardly any monocyte and T-cell infiltration and primarily stimulates microglia activation [189, 190]. In fact, using mice that lack both T cells and B cells (Rag-1^−/−^ mice), it was shown that CD4^+^ and CD8^+^ T cells are required for successful remyelination upon lysolecithin-induced demyelination [191], while T cells or B cells are not essential for cuprizone-induced de- and remyelination [192]. Studies with these experimental de-and remyelination models revealed that successful remyelination depends on OPCs adjacent to the injured area to be transcriptionally activated, followed by their proliferation and migration towards the demyelinated area, and subsequent differentiation of the recruited OPCs to mature myelinating oligodendrocytes [10]. In addition, microglia and astrocytes are recruited to the lesioned area upon toxin-induced demyelination [187, 193].

Microglia, the resident immune cells of the CNS, are one of the first responders upon demyelination: they initiate an innate inflammatory response and clear myelin debris [194]. Microglia responses are very heterogeneous and complex. Different, not yet fully defined activation states exist, of which the classical pro-inflammatory and alternative regenerative phenotype are the most studied [195–198]. Transcriptomic analysis of isolated microglia at different stages upon cuprizone-induced demyelination shows a signature that supports remyelination already at the onset of demyelination involving, among others, phagocytosis of myelin debris [199]. Clearance of degenerated myelin is essential for remyelination, as myelin proteins are known to negatively influence remyelination by inhibiting OPC differentiation [200–202].

Similarly, depletion of specific microglia/macrophage phenotypes in toxin-induced demyelination models demonstrates that while the pro-inflammatory phenotype is initially required, induction of an anti-inflammatory regenerative phenotype of microglia/macrophages is essential for effective remyelination [203]. To control clearance of myelin debris and to accomplish remyelination, bilateral cross-talk between microglia with other CNS (glia) cells is of utmost importance. Microglia promote astrocytic activation [204, 205] and modulate OPC differentiation, while astrocytes instruct microglia and OPCs [106, 206, 207]. To control demyelination and to obtain remyelination, astrocytes play a dynamic and active role. They enhance the immune response by releasing cytokines and chemokines that recruit microglia to the lesion site, inhibit demyelination by releasing anti-inflammatory cytokines and regulate myelination by transiently depositing distinct extracellular matrix molecules that guide OPC proliferation, migration and differentiation [106, 107, 178, 207, 208]. Also, astrocyte ablation delays myelin debris clearance [193], what is required for remyelination to occur [200]. Also, it inhibits the regeneration of oligodendrocytes and myelination [193]. In analogy to microglia, distinct astrocyte phenotypes exist, A1 astrocytes being the harmful type and A2 astrocytes that upregulate neurotrophic factors being protective [209]. Classically (LPS) activated microglia, via the secretion of IL1-1α, TNF and C1q, are required to generate A1 astrocytes in vivo [209]. This suggests a strong interplay between microglia and astrocytes from the onset of CNS injury onwards, concomitantly with the axon-derived secreted and adhesive factors.

### Interplay of astrocytes and phagocytosing cells via galectins

**Fig. 2:**
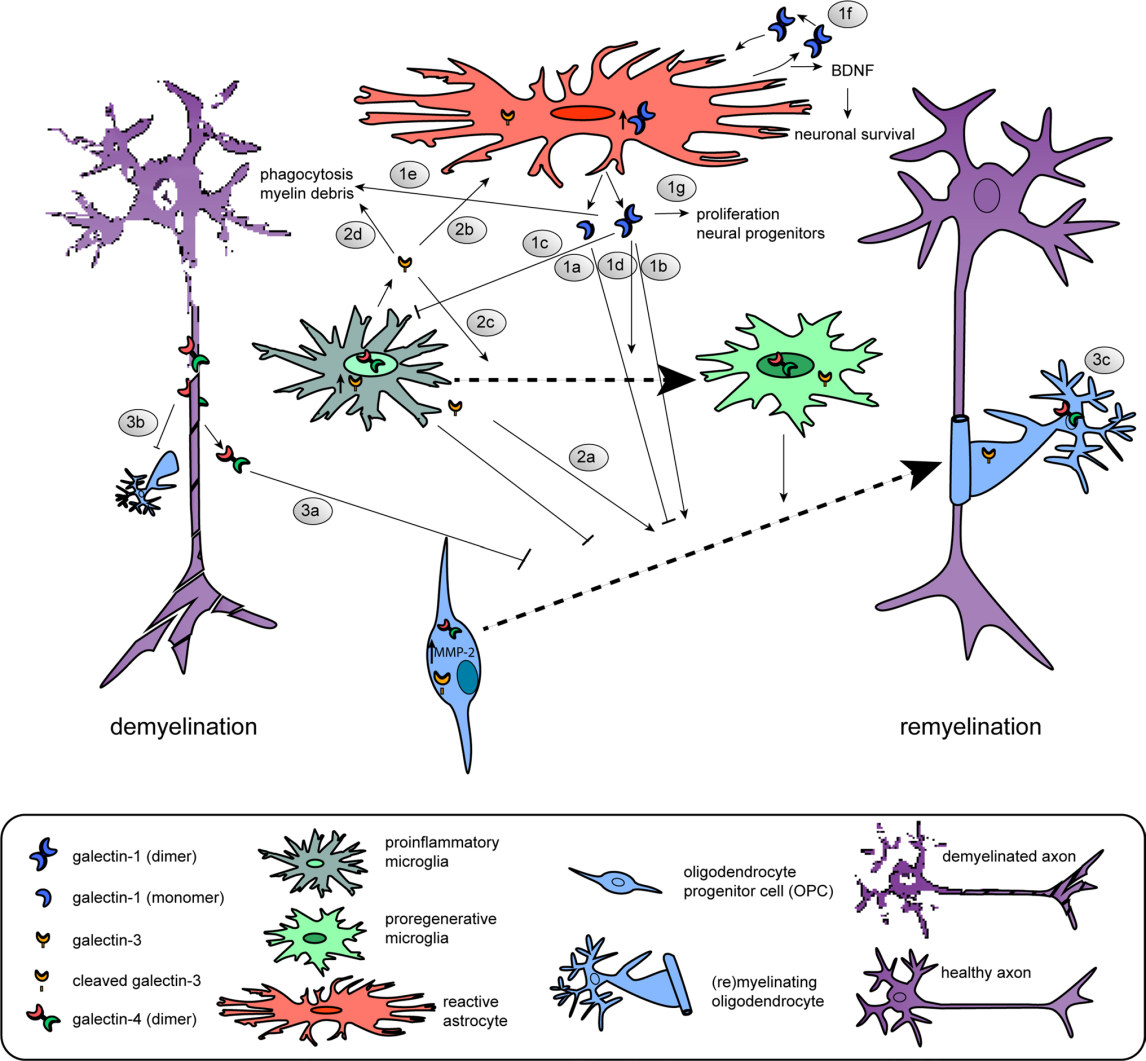
Schematic illustration of the cellular expression and role of galectins-1, -3 and -4 in the regulation of OPC differentiation upon successful remyelination. **1** Galectin-1 is mainly expressed and secreted by (reactive) astrocytes. Low galectin-1 levels (likely mainly monomeric) impair OPC differentiation (**1a**, [156], whereas high levels of galectin-1 (likely mainly dimeric) increase OPC differentiation (**1b**, [158]). Galectin-1 binds to classically activated microglia and inhibits their polarization towards a pro-inflammatory phenotype (**1c**, [212]), accelerates the shift towards an alternatively activated pro-myelinating microglia phenotype (**1d**, [156]), and increases their capacity to phagocytose remyelination-inhibiting myelin debris (**1e**, [156]). Via a positive feedback loop [212], galectin-1 stimulates the release of BDNF by astrocytes (**1f**, [217, 218]) and enhances the proliferation of neural progenitors (1**g**, [219]). **2***Galectin*-*3* is expressed by microglia and oligodendrocyte lineage cells. Oligodendroglial galectin-3 is processed by MMP-2 shortening its N-terminal tail in OPCs, but not mature oligodendrocytes. Galectin-3 treatment promotes OPC differentiation (**2a**, [123]), may regulate astrocyte responses (**2b**, [221], favors polarization to pro-regenerative microglia (**2c**) and increases phagocytosis of myelin debris by microglia (**2d**, [225]). **3***Galectin*-*4* is re-expressed by neurons and considered to be transiently released by axons to negatively regulate the differentiation of OPCs (**3a**, [179]). In addition, the galectin-4-containing domains on axons may impede the deposition of myelin (**3b**, [134]). Upon OPC differentiation, oligodendroglial galectin-4 regulates MBP promoter activity (**3c**, [148]). Galectin-4 is present in the nucleus and/or cytosol of microglia. The underlying mechanism(s) of action of galectins-1, -3 and -4 upon de-and remyelination is (are) summarized in Table 1

As galectins are known to be involved in neuroinflammation and both endogenous and exogenous galectins modulate developmental myelination, the expression and function of galectins upon demyelinating injury and subsequent remyelination have been studied both in toxin-induced animal models and in cellular processes relevant to remyelination (Table 1, Fig. 2). Galectin-4 is transiently re-expressed on axons upon cuprizone-induced demyelination ([179], Fig. 2.3). Although no functional studies on the role of galectin-4 upon demyelination and remyelination are available, it is tempting to suggest that similar to the situation in CNS development re-expressed axonal galectin-4 may be involved in the timing of remyelination preventing premature OPC differentiation upon demyelination (Fig. 2.3a) and myelin deposition (Fig. 2.3b). Remarkably, and in contrast to developmental myelination, galectin-4 resides also in the nucleus of microglia/macrophages upon cuprizone-induced myelination ([179], Fig. 2). In vitro analysis revealed that galectin-4 is not secreted by microglia and macrophages [179]. In addition, galectin-4 protein expression is upregulated in cultured alternatively activated microglia and macrophages and present in both the cytoplasm and nucleus, suggesting that it may add to their pro-regenerative properties. In addition, galectin-4 reduces cytokine secretion of anti- and pro-inflammatory cytokines, IL-10 and TNFα in T cells [210]. On the other hand, galectin-4 administration to macrophages increases the secretion of TNFα and IL-10 [211]. This indicates that in different cell types galectins are able to induce a distinct cytokine secretion signature, which may result in different pathways detrimental or supporting remyelination. Of note, context-dependent effects of galectins reflect their ability of binding to different counterreceptors in different cells, a hallmark of their functional versatility.

Functional studies to determine a role of exogenous galectin-1 in remyelination have been performed. Intracranial administration of galectin-1 a few days after lysolecithin-induced demyelination resulted in reduced demyelination and extensive remyelination [156]. In this model, galectin-1 accelerates the shift towards an alternatively activated pro-regenerative microglia phenotype and increases the cell’s capacity to phagocytose remyelination-inhibiting myelin debris ([156], Fig. 2.1c–e). Galectin-1 binds with increased affinity to classically activated microglia and deactivates this detrimental status by retaining the glycoprotein CD45 via lattice formation on the surface, the homodimer being ideal for cross-linking. This way, the phosphatase activity of CD45 is prolonged, which favors alternative polarization [212]. In addition, it has been suggested that galectin-1 may actively promote alternative activation of microglia by binding to neuropilin-1 (NRP-1) [213]; NRP-1 ablation in microglia fails to polarize to the anti-inflammatory phenotype [214] and galectin-1 promotes axonal regeneration upon spinal cord injury by blocking the binding of Sema3A to NRP-1/PlexinA4 complex [215]. Next to galectin-1-mediated acceleration of the shift from classical to alternative microglia polarization, galectin-1 also directly acts on cells of the oligodendrocyte lineage upon lysolecithin-induced demyelination [156], Fig. 2.1a, b). Although the underlying mechanisms remain to be explored, in analogy to neurons, galectin-1 may interfere with Sema3A binding known to prevent OPC differentiation and remyelination [216].

Bringing astrocytes into play, microglial activation is controlled by astrocytes via galectin-1 secretion. In vitro stimulation of astrocytes by anti-inflammatory signals IL-4 and TGFβ1 and galectin-1 itself led to an increase in the release of galectin-1, suggesting a positive feedback loop ([212], Fig. 2.1f). Notably, exogenously supplied galectin-1 reduces the astroglial response upon lysolecithin-induced demyelination [156]. Moreover, recombinant galectin-1 reduces astrocyte proliferation and induces their differentiation with its glycan-binding activity through the activation of protein tyrosine phosphatase [217]. This is accompanied by enhanced production of brain-derived neurotrophic factor (BDNF) [217, 218], a neuroprotective factor which is known to promote neuronal survival and neuronal development (Fig. 2.1f). Upon other types of CNS injury, galectin-1 is prominently expressed and secreted by astrocytes and enhances proliferation of neural progenitors ([219], Fig. 2.1g). Hereby, beneficial effects of exogenous galectin-1 at demyelinating conditions are established, an advantage for considering testing the lectin for a therapeutic potential.

In addition to galectin-1, galectin-3 also exerts different functions in the process of remyelination. For example, *Lgals3*^−/−^ mice show a similar degree of susceptibility to cuprizone-induced demyelination as wild-type mice, but have an impaired efficiency of remyelination, as reflected by an increase in number of collapsed axons with defective myelin wraps [151]. In more detail, OPCs in cuprizone-induced demyelinated areas in *Lgals3*^−/−^ mice are morphologically less complex and have a decreased ability to differentiate, likely due to the absence of exogenous galectin-3 to organize actin cytoskeletal rearrangements ([123, 167], Fig. 2.2a). In contrast, in another study, during cuprizone-induced demyelination in *Lgals3*^−*/*−^ mice, OPC maturation is not affected by the loss of galectin-3 [220]. This may be related to a difference in the way the knock-out mice were generated. The *Lgals3*^−/−^ mice that show perturbed remyelination have an inactivated galectin-3 gene that lacks an exon that encodes a part of the CRD [221, 222], while the *Lgals3*^−/−^ mice that showed no effect on OPC maturation also lacked exons that are required to initiate translation and encode for the N-terminal region of galectin-3 [220, 223]. Intracranial administration of MMP-processed or full-length galectin-3 in cuprizone-fed mice may resolve whether galectin-3 is indeed beneficial for remyelination. This is conceivable, as—seen in the cuprizone model—galectin-3 expression is increased and expressed in microglial cells, but not in astrocytes, and remains high at remyelinating conditions [199], modulating their microglial phenotype ([221], Fig. 2.2c). In addition, in cuprizone-fed *Lgals3*^−/−^ mice astrocytes are more hypertrophic in demyelinated lesions, also suggesting a role for (microglia) galectin-3 in regulating astroglial responses upon demyelination ([221], Fig. 2.2b). The induction of transient and focal ischemic injury in *Lgals3*^−/−^ revealed that galectin-3 is indeed required for injury-induced microglial activation [169]. In contrast, neonatal *Lgals3*^−/−^ mice were protected from hypoxic–ischemic brain injury [185], indicating different means of galectin-3 to modulate microglial phenotype in the adult and immature brain. Another study has demonstrated that during cuprizone-induced demyelination the presence of MMP-3 is increased and that galectin-3 is necessary to upregulate MMP-3 expression and to promote microglial activation [151]. Also, and in contrast to what is observed upon ischemic injury [169], galectin-3-deficient microglia become more proliferative upon demyelination [151]. Of importance now is to resolve whether the actions of microglia-derived galectin-3 upon demyelination are dependent on lectin binding or its non-lectin activities.

A critical part during toxin-induced demyelination is clearing the remyelination-inhibiting myelin debris by resident microglia cells [200]. Galectin-3 is involved in myelin phagocytosis mediated by the Ras/PI3K signaling pathway ([224, 225], Fig. 2.2d) and by regulation of the expression of the phagocytic receptor TREM-2b [221]. Upon demyelination in *Lgals3*^−/−^mice, TREM-2b is not detected on microglia, along with the absence of the activation marker CD68. In addition, *Lgals3*^−/−^mice were also unable to increase TNFα levels upon cuprizone treatment [221], while the mRNA levels of chemokine CCL2, a marker for classically activated microglia, remained high. Altered microglia activation in *Lgals3*^−/−^mice is also reflected by increased levels of caspase-3 activation [221], a marker for apoptosis, in microglia, indicating an anti-apoptotic role of galectin-3. While it is tempting to conclude that galectin-3 may favor polarization towards alternatively activated microglia, another study showed that the addition of galectin-3 to cultured microglia increased the expression of pro-inflammatory cytokines and enhanced the phagocytic capacities of the cells by activating the JAK-STAT cascade [226]. Also, galectin-3 is required for complete activation of TLR4 to initiate TLR4-mediated responses in microglia and for prolonging the inflammatory response [227]. This further complicates the effect of galectin-3 on microglia activation and function, suggesting that a distinct spatiotemporal course of expression of galectin-3 is required for the induction of the correct microglia phenotype to attain successful remyelination. Worth considering, posttranslational modifications such as phosphorylation and the dissection of biological functions via the non-lectin part or CRD may help understand the molecular basis for the contrasting effects of galectin-3 on microglia function.

In summary, galectins-1, -3 and -4 via their interactions act as communication cues between neurons, astrocytes, microglia and OPCs and modulate cellular responses during de- and/or remyelination (Tables 1, 2, Fig. 2). In addition, intimately regulated spatiotemporal expression and secretion of galectins are essential for regulating innate immune responses required for successful remyelination. As consequence, dysregulation in galectin action may contribute to MS pathology. This topic will be discussed next.

**Table 2:** Galectins in non-MS-related CNS injuries

Galectin	Model	Main result	Mechanism	References
In vivo				
gal-1	Spinal cord injury (treatment)	Promotes axonal regeneration in *Lgals1*^−*/*−^ C57BL/6 mice (only dimeric form)	Inhibits Sema3A binding to NRP-1–PlexinA4 complex	[215]
gal-1	Epileptic seizure model (*Lgals 1*^−*/*−^ 129 P3/J mice)	Reduced proliferation of neural progenitors	Astrocyte-secreted gal-1 may act as a growth-stimulating factor and/or increase the supply of neurotrophic factors	[219]
gal-1 gal-3	Stab wound injury (*Lgals 1*^−/−^*Lgals3*^−*/*−^ C57BL/6 mice)	Reduced reactive astrocyte proliferation and their NSC potential	May regulate cell cycle progression at the G1–S-phase transition	[322]
gal-3	Acute ischemia (gal-3 null mutant C57Bl/6 mice)	Defective microglia activation and decreased proliferation	Required for the induction of an TLR2 response, binds to IGFR and essential for IGF1-mediated proliferation	[169]
gal-3	Neonatal hypoxia–ischemia (*Lgals3*^−*/*−^ SV129 mice)	Protected from injury particular in male mice	Increased accumulation of microglia, decreased levels of MMP-9 and less oxidative stress in the absence of gal-3	[323]
gal-3	Severe transient forebrain ischemia (male Mongolian gerbils)	Increased galectin-3 expression in microglia after the onset of neuronal damage in the hippocampal CA1 region	Not a trigger of neuronal death, hypothermia prevents gal-3 expression	[324]
gal-3	Spinal cord injury (*Lgals3*^−*/*−^ C57BL/6 mice)	Increased neurological recovery	Sustains a pro-inflammatory microglia/macrophages phenotype	[281]

*CA* cornu ammonis (hippocampus), *CNS* central nervous system, *gal* galectin, *Iba-1* ionized calcium-binding adaptor molecule 1, *IGF* insulin-like growth factor, *IGFR* insulin-like growth factor receptor, *MMP* matrix metalloproteinase, *NP-1* neuropilin-1, *TLR2* Toll-like receptor 2

## Galectins in MS pathology

### MS pathology: a role of peripheral and resident cells

Neurological diseases that involve myelin pathology can be divided into inherited or acquired disorders (reviewed in [228, 229]). Leukodystrophies are hereditary myelin disorders that are characterized by either hypomyelination or demyelination. Strikingly, the primary affected cell type in leukodystrophies does not have to be the oligodendrocyte itself, i.e., the genetic defect may also cause dysfunction of astrocytes or microglia, emphasizing the role of other glial cells in myelin biogenesis. Next to genetic factors, viral, trauma (ischemic brain injury), toxic, metabolic and immune-mediated factors also play a role in the etiology of demyelination. MS has been known to be the archetypal acquired demyelinating disorder of the CNS. The cause of MS is unknown, although both environmental exposure and genetic susceptibility appear to play a role. MS is characterized by inflammation, demyelination, axonal damage and (astro)gliosis and manifests as demyelinated lesions at multiple regions in the brain and spinal cord [3]. Autoreactive pathogenic peripheral CD4^+^ helper T cells penetrate the BBB, are re-activated in brain parenchyma by CNS-associated antigen-presenting cells, and play a central role in the development of demyelinated lesions in RR-MS. The disease pathogenesis during RR-MS is driven by the fine balance between Th1 and Th17 cells, and their suppressive regulatory T cells (Tregs). These myelin-reactive peripheral cells cross the BBB and mediate myelin degeneration [230–234]. Peripheral monocyte-derived macrophages are also recruited to demyelinated lesions [235–237]. In active MS lesions, it is estimated that 55% of the macrophages arise from infiltrated monocytes [238]. In contrast, only a few peripheral macrophages are present in cuprizone-induced demyelinated lesions [190]. Infiltrated macrophages will add microglia to resolve the inflamed and demyelinated area, while differential functions are apparent, microglia being more supportive and macrophages more immune reactive [196, 239–241]. Interestingly, microglia and macrophages directly communicate with each other. This has recently been shown in a model for spinal cord injury, where infiltrated macrophages reduce microglia-mediated phagocytosis and inflammatory responses [242]. However, it is not fully understood whether the infiltration of peripheral cells is a primary autoimmune response or a secondary response to demyelination [3, 243–245], as primary degeneration of axons is also a characteristic feature of MS [246]. In fact, de-adhesion of the inner loop of myelin to the axonal surface has been postulated to be the initial event in MS lesion formation [245, 247].

Although spontaneous remyelination occurs, most commonly at early stages of MS and in active lesions, a major cause of the neurological deficits and disease progression is due to incomplete or failed remyelination, particularly at the later progressive MS stage and in chronic lesions [9, 10, 15, 239, 241, 243]. Remyelination is, however, observed in some patients at late-stage progressive MS, emphasizing the heterogeneity in MS pathology [3, 248, 249]. The factors involved in remyelination failure are many, including axonal damage, dysregulation of the cellular and molecular microenvironment within the lesions and/or failure of OPC recruitment. Strikingly, post-mortem analysis revealed that in approx. 70% of MS lesions OPCs are present [13, 15, 16], indicating that extrinsic and/or intrinsic factors in MS lesions that allow differentiation are derailed. During the active phase of an MS lesion, microglia and macrophages are skewed towards a pro-inflammatory phenotype [250, 251]. However, given the altered environmental factors in MS lesions at hand, a major subset of infiltrated macrophages and resident activated microglia acquire eventually an intermediate activation status [252, 253]. As an anti-inflammatory regenerative phenotype of microglia and macrophages is essential for effective remyelination [203], dysregulated activation of microglia and/or macrophages may contribute to remyelination failure in MS. Also, reactive astrogliosis and astrocytic scar formation negatively affect OPC recruitment and differentiation, and thereby remyelination [254], but are on the other hand also beneficial for functional CNS recovery [106, 207, 255, 256].

Dysfunction of astrocytes and/or microglia, for example, dysregulates galectin expression and secretion, disturbs their interplay, leading to a molecular environment that is non-permissive for OPC maturation. Increased expression of galectins-1, -3, -4, and -9 in CNS-resident cells is apparent in MS lesions compared to control white matter [257, 258], and galectin-1 is one of the most upregulated genes in MS-associated microglia signature [259]. Galectins are also regulators of peripheral immune responses [40, 258, 260] and given the infiltration of peripheral cells in MS lesions, galectins present in the periphery may (indirectly) contribute to remyelination failure. Indeed, next to infiltration of macrophages, infiltrating regulatory T cells have regenerative properties, by promoting OPC differentiation and remyelination [181], while Th17 cells decrease OPC differentiation and survival [261]. Therefore, before discussing whether an increased presence of these galectins in MS lesions is beneficial or detrimental to remyelination, we first describe whether these galectins, when present in the periphery, may be involved in adaptive immune responses in MS.

### Galectins in MS-related neuroinflammation

Experimental models that recapitulate all aspects of MS pathology are not available, in part due to the unknown cause, if only one, and heterogeneity in MS. While in toxin-induced demyelination models pathogenic T cells are not involved in the demyelination process [191, 192], the adaptive immune system plays an important role in inducing demyelination in experimental autoimmune encephalomyelitis (EAE) models. Depending on the species, strain, and the used myelin protein/peptide, different courses, including acute, relapsing-remitting and chronic, can be initiated in EAE models [262]. The initiation and peak of the disease are mediated by Th1 and Th17 responses, while recovery from EAE is initiated by a shift towards Th2 cell responses [263], although another study found that Th2 cells also have the potential to induce EAE [264]. Furthermore, in the EAE model by controlling cytokine production and the movement of T cells, regulatory T cells have been found to be protective and mediate recovery from EAE [265, 266]. Also, as in MS lesions, infiltrated peripheral macrophages, as well as B cells, are present at the affected areas [267]. In contrast, the role of microglia in EAE is considered to be less important than in MS [243]. Therefore, the EAE model is indispensable in MS research and also exploited to elucidate the role of galectins in modulation of inflammatory response in the CNS (Table 3).

**Table 3:** Galectins during MS-relevant inflammation

Galectin	Model	Main result	Mechanism	References
In vivo				
gal-1	EAE (GP-BP, female Lewis rats, treatment before or at induction)	Inhibits clinical and histological signs, most effective when applied at induction	Prevents sensitization of encephalitogenic GP-BP-specific T cells and induces timely expression of suppressor CD8 + T cells	[268]
gal-1	EAE (MOG_35−55_, female* Lgals1*^−*/*−^ 129/Sv mice)	Exacerbated disease severity	Increases pathogenic Th1 and Th17 responses	[269]
	EAE (MOG_35−55_, C57Bl/6 mice, treatment after immunization but before disease onset)	Ameliorates disease severity	Reduces the numbers of IL-17 and IFNγ-producing CD4^+^ T cells	
gal-1	EAE (MOG_35−55_, female* Lgals1*^−*/*−^ C57Bl/6 mice)	Not reported	Enhances classical microglia activation, promotes axonal damage	[212]
	EAE (MOG_35−55_, female* Lgals1*^−*/*−^ C57Bl/6 mice, adoptive transfer WT astrocytes)	Ameliorates disease severity	Regulates microglial activation	
	EAE (MOG_35−55_, female C57Bl/6 mice, treatment around onset clinical disease)	Ameliorates disease severity	Decreases microglial activation, prevents neurodegeneration and demyelination and reduces GFAP expression	
	EAE (MOG_35−55_. female* Lgals1*^−*/*−^ C57Bl/6 mice, adoptive transfer of treated control and LPS-stimulated microglia)	Ameliorates disease severity	Prevents microglia activation	
gal-3	EAE (MOG_35−55_,* Lgals3*^−*/*−^ C57Bl/6 mice)	Slightly delayed onset and ameliorated disease severity	Decreases IL-17 and IFNγ levels, increases the development of Th2 and Treg cells	[272]
gal-4	chronic relapsing EAE (rrMOG_1–125_, in male Dark Agouti rats)	Increased presence in inflammatory infiltrates	Localizes to ED1 + cells at relapse phase	[179]
gal-8	EAE (MOG_35−55_,* Lgals8*^−*/*−^ C57BL/6NTac mice)	Faster onset and increased disease severity	Increases Th17 polarization and decreases the frequency of Treg cells that impact Th17	[288]
	EAE (PLP_139–151_, female C57BL/6 mice, treatment at induction)	Delayed onset and ameliorated disease severity	Apoptotic elimination of activated Th17 cells	
gal-9	EAE (MOG_35−55_, female C57BL/6J mice, treatment after immunization but before disease onset)	Ameliorates disease severity	Eliminates IFNγ producing Th1 cells through Tim3	[282]
	EAE (MOG_35−55_ SJL/J mice, injection at induction)	Exacerbates disease severity		
In vitro				
gal-1	Human bone marrow mesenchymal stem cells (MSCs)	MSC-derived gal-1 inhibits T-cell proliferation	Binds to NP-1 on T cells	[213, 271]
gal-1	Primary microglia (C57BL/6 WT and* Lgals1*^−*/*−^ C57Bl/6 mice mice, treatment)	Deactivates classically activated microglia	Controls microglial activation through p38MAPK, CREB and NF-κB signaling pathways and promotes microglial deactivation by retaining CD45 at the surface	[212]
gal-3	Blood monocyte-derived human macrophages	Gal-3 expression and proteolytic processing are higher in alternatively activated cells, while its secretion is higher in classically activated macrophages	Not determined	[280]
gal-3	Microglia and astrocytes (primary cells, Sprague–Dawley rats, BV2 microglia cell line, treatment)	Enhances production of pro-inflammatory mediators	Triggers the JAK-STAT signaling cascade through IFNRG1(CRD-independent, IFNγ-independent)	[226]
gal-3	Bone marrow- and blood monocyte-derived macrophages (129Sv WT and* Lgals3*^−*/*−^ mice, THP-1 monocytic cell line)	Reduced alternative macrophages activation	Mediates alternative activation by PI3K activation upon binding to CD98	[279]
gal-9	Primary microglia, astrocyte and mixed glial cultures (Sprague–Dawley rats, C57Bl/6J WT and* Lgals9*^−*/*−^ mice)	Astrocyte-derived gal-9 enhances microglia TNF production	Tim-3 independent	[299]
		poly(I:C-) treated microglia stimulate gal-9 mRNA expression in astrocytes	Mediated via a heat-sensitive microglia secreted factor	

*EAE* experimental autoimmune encephalomyelitis, *gal* galectin, *GFAP* glial fibrillary acidic protein, *GP-BP* guinea pig myelin basic protein, *IL* interleukin, *LPS* lipopolysaccharide *MOG* myelin oligodendrocyte glycoprotein, *MSC* mesenchymal stem cells, *NP-1* neuropilin-1, *poly(I:C)* polyinosinic:polycytidylic acid, *siRNA* small interfering RNA, *Tim-3*, T-cell immunoglobulin and mucin domain-containing molecule-3, *Th* T helper, *TNF* tumor necrosis factor,* WT* wild-type

Endogenous galectin-1 expression is dynamically regulated in EAE, being increased in astrocytes at the lesion edges, and in subsets of CD4^+^ Th1 cells and microglia before and at the onset of EAE symptoms, while its expression remains increased in astrocytes at the chronic stage [212]. Intravenously administration of galectin-1, either before or at EAE onset, results in a reduced severity of symptoms [268], mainly by inducing tolerogenic dendritic cells, selective elimination of pro-inflammatory Th1 and Th17 cells and enhanced development of Tr1 and regulatory T cells [269, 270]. This is also shown by the inhibitory effect on T-cell proliferation upon binding of galectin-1 to NP-1, a glycoprotein counterreceptor [213, 271]. Consistently, induction of EAE in *Lgals1*^−/−^ mice increases the severity of symptoms via a T helper cell response mechanism and a concomitant increase in classically activated microglia and axonal damage [270]. Moreover, adoptive transfer of galectin-1-secreting astrocytes or galectin-1-treated microglia augmented EAE symptoms via a mechanism that involves deactivation of pro-inflammatory microglia [212]. This indicates a role of this lectin as an anti-inflammatory mediator and neuroprotective agent.

*Lgals3*^−/−^ mice show reduced severity upon induction of EAE [272], a sign for a detrimental role for galectin-3 in EAE pathology. Interestingly, this effect is associated with a decreased Th17 and an increased regulatory T-cell response, i.e., an underlying mechanism similar as observed for galectin-1 administration (see above), as well as decreased infiltration of peripheral macrophages [272]. In contrast, a higher incidence and more severe course of EAE is apparent in mice lacking Mgat5, an enzyme necessary for β1,6 branching (GnT-V) on *N*-glycans, to which galectin-3 can bind, preferably when presenting LacNAc repeats. Given the hereby caused reduction in galectin-3 counterreceptors on the T-cell surface, *Mgat5*^−*/*−^ mice displayed enhanced T-cell receptor (TCR) clustering and diminished polarization to Th2 cells, and developed spontaneous inflammatory demyelination and neurodegeneration [273, 274]. Similarly, earlier studies have identified galectin-3 as a negative regulator of T-cell activation [273, 275]. By cross-linking TCRs and other glycoproteins on the surface of naive T cells, galectin-3 restricts TCR clustering at the site of antigen presentation, which prevents T-cell activation. Thus, the role of galectin-3 in T-cell responses in EAE is currently controversial.

Inside the CNS, galectin-3 is highly implicated in the pathophysiology of EAE. In EAE, galectin-3 is present in phagocytosing microglia and macrophages and is upregulated in areas of demyelination and myelin degeneration [276, 277]. Along with the expression of MAC-1 (CD116), which mediates myelin phagocytosis, galectin-3 (also known as MAC-2) is, as in the case for microglia, an in vivo marker for an activated phagocytosing macrophage [276, 278]. Interestingly, peripheral macrophages obtained from *Lgals3*^−/−^ mice are defective to become alternatively activated [279]. Alternatively induced macrophages have increased expression and secretory activity for endogenous galectin-3 [279]. On macrophages, galectin-3 binds to CD98 and stimulates the PI3K pathway that drives the alternative activation route of macrophages [279]. Hence, this type of macrophage activation phenotype is dependent and sustained by the endogenously expressed galectin-3. In contrast, in vitro, enhanced secretion and expression of galectin-3 by classically activated human macrophages are observed [280]. Moreover, galectin-3 sustains a pro-inflammatory microglia and macrophage phenotype in a spinal cord injury model [281]. Evidently, effects of galectin-3 on phagocytosing cells are complicated, suggesting that a timed and context-dependent expression of galectin-3 is necessary for the induction of the correct microglia/macrophage phenotype. Despite the dual properties of galectin-3 in EAE, i.e., peripherally and at the lesion site, the overall impact upon demyelination in the CNS is that galectin-3 is instrumental in modulating the phenotype of macrophages at the demyelinated area.

Galectin-9, a tandem-repeat-type family member, has also been implicated in EAE development. Intraperitoneal administration of galectin-9 early after EAE induction results in a reduced severity, whereas siRNA-mediated silencing of galectin-9 results in an increased severity of clinical symptoms [282]. Galectin-9 is a binding partner for the glycoprotein Tim-3, a type-1 membrane protein specifically expressed on the surface of fully differentiated Th1 cells. Galectin-9 is a negative regulator of Th1 cell function and induces phosphorylation of Tim-3 which in turn triggers Th1 cell apoptosis, thereby shifting the balance towards Th2 cells and reducing extent of inflammation [282, 283]. Bat-3, a binding partner of intracellular tail of Tim-3, promotes proliferation and is a protective agent of Th1 cells against galectin-9-mediated cell death [284]. Also, reduced expression of Tim-3 on T cells has been suggested as an intrinsic defect that contributes to the pathogenesis of MS [285]. Blocking the interaction of galectin-9 with Tim-3 results in reduced apoptosis of T cells of RR-MS patients, but not in T cells obtained from PP-MS patients, which may relate to the upregulation of Bat-3 in PP-MS [286]. Thus, the Tim-3/Galectin-9 pathway seems to be malfunctional in PP-MS. Of note, when IFN-β, applied to treat RR-MS, is fused to galectin-9 to build a conjugate, the immunosuppressive effects of IFN-β on Th1 cells are more effective and its side effects are reduced [287]. Galectin-8 that also has the tandem-repeat-type architecture exerts similar immunosuppressive responses as galectin-9 does in EAE. Fittingly, EAE is exacerbated in *Lgals8*^−/−^ mice, by modulating the balance of Th17 and Th1 cells and their Tregs [288]. This galectin induces apoptosis in Th17, but not Th1 cells, and galectin-8 administration ameliorates EAE [288]. In the clinical situation, the possibility for the occurrence of auto-antibodies against galectins should be considered (see below, [289, 290]).

In conclusion, galectins-1, -8 and -9 exert immunosuppressive and anti-inflammatory effects, and galectin-3 acts as a pro-inflammatory regulator. These observations strongly suggest that galectins-1, -3, -8 and -9 are involved in EAE/MS pathology by modulating T-cell-mediated inflammation, macrophage recruitment and function at the periphery and/or within the infiltrated lesioned areas. While the effect of galectin-4 in EAE pathology has not been examined, the ameliorating effect of sulfatide treatment on EAE (sulfatide being a counterreceptor), among others via inhibition of T-cell proliferation, is galectin-4 dependent [291]. It is obviously of importance to gain further knowledge on how these galectins play a role in remyelination failure, which mainly unfolds inside the CNS, and then how this knowledge can be used to overcome remyelination failure in MS.

### Galectins in remyelination failure

Although OPCs are recruited at the demyelinated site, an environment that negatively impacts OPC differentiation is suggested to be one of major causes of remyelination failure in MS [9, 10]. Why remyelination ultimately fails in MS is still unknown and may not be assigned to one particular cause, but is likely related to multiple events in different cells and even may differ in the different type of MS lesions. MS lesions can be partly remyelinated, but remyelination is most prominent in sites of active lesions [249, 292, 293], suggesting that the molecular and cellular environment is important for remyelination efficiency. This may include a beneficial role for microglia/macrophages, when appropriately activated, and a detrimental role for the astrogliotic scar in chronic lesions [292, 294]. Given their role in remyelination, dysregulated galectin expression and/or function may contribute to remyelination failure in MS.

Galectin-1,-3, and -9 protein levels are significantly increased in MS lesions compared to control white matter [167, 258], a clear hint of the contribution to MS pathology, including remyelination failure. In MS lesions, galectin-1 is mainly localized to the cytoplasm of microglia/macrophages, while the number of astrocytes harboring galectin-1 is decreased in astrocytes compared to control white matter [258]. In contrast to cuprizone-mediated demyelination, microglia/macrophages rather than astrocytes appear as main cellular source of galectin-1 in MS. Studied by western blot analysis in vitro, MS astrocytes externalize relatively more galectin-1 as homodimers, while normal astrocytes mainly secrete monomers [258]. In addition, in contrast to normal astrocytes, galectin-1 is localized in nuclei of MS astrocytes, both in vitro and in vivo [258]. This may be a feature of A1 astrocytes, which are observed in lesions of RR-MS patients [209], while their role in remyelination (failure) remains to be established. Presence of galectin-1 as homodimers may be beneficial for OPC differentiation when ligated to galectin-1-binding sites on OPCs [156, 295], while galectin-1 monomers secreted by normal astrocytes may prevent OPC differentiation [123]. Also, galectin-1 deactivates classically activated microglia, thereby favoring their alternative activation [212] and inducing myelin phagocytic capacity, both processes beneficial for OPC differentiation [200, 203].

Galectin-3 expression is increased in active MS lesions, and the lectin is present in the cytoplasm of microglia/macrophages and astrocytes [258]. At first glance, increased galectin-3 expression seems to be beneficial for OPC differentiation, as exogenously applied galectin-3 directly promotes OPC differentiation [123], and indirectly modulates OPC differentiation by playing a pivotal role in the switch towards a regenerative phenotype of microglia/macrophages [225, 276]. Nevertheless, although more frequently observed in active lesions compared to other MS lesions, complete remyelination fails in MS lesions. This may imply that (1) galectin-3 is not secreted at the lesion site, (2) exogenous galectin-3 is not sufficient to induce OPC differentiation, i.e., other local inhibitory factors may be dominant, or (3) OPCs and/or microglia/macrophages may lack cognate determinants for galectin-3 to initiate a response.

Although not observed at the mRNA level [258], axonal galectin-4 is (re)-expressed in chronic MS lesions, as also observed in cuprizone-induced demyelination and likely a default response to demyelination [179]. Provided that axonal galectin-4 is secreted in MS lesions, galectin-4 may impair OPC differentiation [179]. Its presence in axons per se likely prevents myelination deposition [134]. In active MS lesions, galectin-4 was observed in the nucleus and cytoplasm of activated microglia/macrophages, which efficiently endocytose galectin-4 in vitro [179]. In this way, microglia/macrophages may scavenge galectin-4 away from immature oligodendrocytes and attenuate the negative role of galectin-4 on differentiation [179]. Its persistent presence in and potential secretion by demyelinated axons indicate that axonal galectin-4 in MS lesions may be a potential cause of remyelination failure in MS, particularly in chronic lesions where the number of microglia/macrophages is reduced [296, 297].

The role of galectins-8 and -9 in demyelination–remyelination models has not been thoroughly investigated yet. Although total galectin-8 protein expression is not enhanced, at the cellular level galectin-8 is abundantly present in microglia/macrophages in active MS lesions, while being absent in microglia in tissue adjacent to the lesion [258]. The presence of galectins-8 and -9 at the lesion site may have immunomodulating roles on the infiltrated T cells that are present in MS lesions, which may include selective apoptosis of Th17 and Th1 cells. Th cells and cytotoxic T cells are localized perivascularly, as well as being diffusely present throughout MS lesions [292, 298]. Galectin-9 is produced and secreted by activated astrocytes [299–301] and synergizes with the TLR3 agonist poly(I:C) to increase TNF-α secretion by microglia in a Tim-3-independent manner [299]. Interestingly, poly(I:C)-stimulated microglia enhance galectin-9 expression in astrocytes, emphasizing a role for galectin-9 in astrocyte–microglial cross-talk. Although galectin-9-specific mRNA levels are increased in astrocytes that were isolated from MS lesions [257], enhanced protein expression of galectin-9 in MS lesions is related to its presence in microglia/macrophages rather than astrocytes [258]. Remarkably, galectin-9 is mainly present in microglia/macrophage nuclei in active lesions, while in chronic active MS lesions galectin-9 exclusively localizes to the cytosol [258]. This nuclear-to-cytoplasmic relocation may reflect changes in galectin-9 function, as respective shuttling of galectin-3 is known to exert [66]. A potential role of endogenous galectin-9 in microglia/macrophages polarization and secretion of galectin-9 by microglia/macrophages remain to be determined. Notably, galectin-9 levels are higher in CSF of SP-MS patients than in CSF of RR-MS patients, which is likely a reflection of innate rather than adaptive immune responses [302]. This indicates that galectin-9 is involved in the pathophysiology of SP-MS, and given its role in interglial communication, may contribute to remyelination failure by retaining or inducing a pro-inflammatory microglia/macrophage phenotype.

In summary, galectins-1,-3, -4, -8 and -9 may contribute to remyelination failure in MS lesions. Although several questions remain open on the contribution of galectins to remyelination failure, the fact that several galectins appear to be involved in successful remyelination combined with the derailed regulation of galectins in MS lesions, investing further efforts to delineate, if possible, a therapeutic potential appears to be warranted.

## Galectins: novel targets or tools to overcome remyelination failure in MS?

A favored strategy to overcome remyelination failure in MS is to promote OPC differentiation. Several compounds, including benztropine, are known to promote remyelination in vivo after cuprizone treatment by direct antagonism of receptors [303]. Although these compounds are very effective in promoting OPC differentiation (reviewed in [304]), OPCs in MS lesions usually face cellular and molecular environments that inhibit their differentiation. Given the multifaceted actions of galectins in interglial communication, there are several hurdles that have to be overcome before considering modifying therapies that are based on the gain or loss of (extracellular) galectin function.

The first is to define whether galectins may be useful to overcome remyelination failure, also taking into account the context of the cellular environment in the distinct MS lesions. Galectins-1 and -3 act as modulators of microglia and macrophage activation and phenotype, driving the onset of remyelination. In addition, galectins-1 and -3 enhance, whereas galectin-4 impairs OPC differentiation. Therefore, in active MS lesions, where microglia and macrophages are evenly distributed throughout the lesion, galectins-1 and -3 may help to skew the intermediate phenotype microglia and macrophages towards an anti-inflammatory phenotype and increase phagocytic capacity [156, 212, 279]. Microglia and macrophages are hardly present in chronic inactive MS lesions, while their presence is limited to (parts of) the lesion border of chronic active lesions [249, 292]. Therefore, targeted delivery of these galectins may promote OPC differentiation in chronic lesions. However, in chronic lesions galectin-4 is also abundantly present, in addition to many other negative regulators of OPC differentiation. Hence, it is essential to critically assess whether galectins-1 and -3 can overcome remyelination failure in a MS lesion environment, and to verify whether a specific galectin-4 antagonist such as a high-affinity sulfatide derivative or a neutralizing antibody against galectin-4 counteracts its inhibiting effect on OPC differentiation.

A second hurdle to be cleared is that the biochemical nature of the binding sites of galectins-1, -3, and 4 on microglia, macrophages, astrocytes, and cells of the oligodendrocyte lineage is not known yet. Identification of their counterreceptors, e.g., being a protein and/or a glycosphingolipid, will provide useful information about the chain of events toward effects. Equally important, although the activity profiles of galectins-1 and -3 appear to be similar, these galectins are not redundant and can likely act via different mechanisms at different sites, as they do not compensate for each other in experimental models. Therefore, it is of utmost importance to define the biochemical nature of counterreceptors for galectins, in the case of galectin-1 especially for both forms. Notably, the glycosylation signature of the putative galectin counterreceptors(s) may alter during development and in different phases of de- and remyelination, and may thus be only transiently suited for the galectin. How access to the termini of *N*-glycans of the α_5_β_1_-integrin is regulated via α2,6-sialylation and sialic acid biosynthesis as crucial molecular switches to enable galectin-1-dependent anoikis induction by the tumor suppressor p16^INK4a^ as master factor provides a salient lesson for such mechanisms [305]. Indeed, glycosylation is subject to dynamic regulation, which definitely influences the extent of lattice formation by galectins [306]. Respective molecular switches with clinically relevant implications have been disclosed in the case of Tn sialylation by ST6GalNAc2 that blocks production of *O*-glycan core 1/2 counterreceptors for galectin-3 and hereby attenuates breast cancer metastasis [307] and in the case of prostate cancer progression, in which *O*-glycan core 2 presence and susceptibility to galectin-1 and sialylated core 1 presence and binding to galectin-4 let the clinical course go in opposite directions [308–310]. Also, oligodendroglial counterreceptors for galectins-3 and -4 are transiently expressed and spatially distributed at the surface [123, 124]. Identification of binding sites of galectins will also provide information whether these surface receptors are present in MS lesions and can thus be targeted by galectins when applied as a tool. As indicated above for the sialylation status of *N*-glycans and also known for enzymatic conversion among gangliosides, i.e., GD1a to GM1, or the shift from core 1 to core 2 *O*-glycans, the glycosylation status of glycoconjugates influences avidity of galectins binding, and disease-related changes in glycosylation may prevent galectin binding. Environmental and genetic factors are shown to dysregulate *N*-glycosylation by Golgi-localized enzymes that could lead to inflammatory demyelination and neurodegeneration in MS [311]. Finally, as the same galectin has multifaceted roles in different cells at different stages of the remyelination process, knowing the identity of the counterreceptor(s) will allow for more specific understanding of post-binding effects.

Galectins are potent modulators of peripherally immune responses and in this way may affect the clinical course of MS [268, 269, 272, 282, 288]. Hence, a third hurdle is that targeted delivery of galectins or their agonists/antagonists to MS lesions is required. For example, modulation of galectin levels may be accomplished by their local secretion at the lesion area from engineered nanocarriers and exosomes that pass the BBB. Also, it is essential to determine their effect on axons and astrocytes in the demyelinated areas. For example, galectin-1 dimers induce neuronal degeneration [162] and promote astrocyte differentiation [217]. On the other hand, the anti-inflammatory properties of galectin-1 may overcome the effect of infiltrated T cells [312, 313]. In addition, the heterogeneity and region-specific differences in microglia, astrocytes and OPCs in, for example, gray and white matter areas also need to be considered, as well as that timely expression of several galectins is crucial for proper remyelination. Hence, targeting galectin–glycan interactions may represent a new therapeutic approach for promoting remyelination in MS lesions, provided that a comprehensive analysis on their effect on all lesion-resident cells has been performed.

Having emphasized the importance of the protein architecture for activity (Fig. 1), it is noteworthy that altering protein design is a means to generate new classes of galectin-based effectors, to block or to induce activities with clinical benefit. Galectin-3, for example, can become a cross-linking homodimer like galectin-1, and galectin-1 a galectin-3-like chimera-type protein [314]. This special type of variant of galectin-1, monomeric in solution in contrast to the galectin-1 homodimer, has been shown to serve as functional antagonist of the wild-type protein [55]. The CRDs of galectins-3 and -4, here especially the N-terminal unit, may likewise serve as galectin-based means to saturate counterreceptors and hereby preclude triggering of harmful effects by the physiologically present galectin. Due to the high target specificity for glycoconjugates of each domain that they share with the wild-type proteins, this engineered type of reagent will likely be preferable to sugar-based inhibitors. Concerning galectin blocking, another means is specific (auto)antibodies, briefly referred to above.

## Autoantibodies against galectins in MS

Autoantibodies against galectins may play an important role in the onset or progression of immune diseases [315, 316], and the presence of autoantibodies could contribute to a continuous immune dysregulation in MS. Furthermore, autoantibodies against galectins may be a possible therapeutic target to treat MS [288, 289, 317], while on the other hand autoantibodies may also interfere with the action of galectins when therapeutically applied. A recent study found that sera from SP-MS patients contain autoantibodies against galectin-3, which may serve as a biomarker for SP-MS [317]. Anti-galectin-3 antibody binding to galectin-3 at the surface of human brain microvascular endothelial cells increases, among others, the expression of ICAM-1 on brain endothelial cells. This contributes to the disturbance of BBB integrity, resulting in leukocyte leakage to the CNS [317]. Interestingly, fingolimod, an oral sphingosine-1-phosphate-receptor modulator used for treatment of RR-MS, leads to reduced level of infiltration of aggressive lymphocytes and was suggested to prevent endothelial cell activation induced by anti-galectin-3 antibodies [317]. Similarly, when administered at the time of EAE immunization, the FDA-approved drug for MS, glatiramer acetate (copolymer 1) reduces galectin-3 expression on macrophages at the lesion area [276]. Anti-galectin-8 antibodies have also been detected in sera and CSF of RR-MS patients: these proteins neutralize the immunosuppressive role of galectin-8 [288] and may thus worsen relapses. Hence, anti-galectin-8 antibodies may hold promise as a potential early prognostic marker. In addition, levels of autoantibodies against galectin-1 were significantly higher in sera from MS patients [289], and it would be interesting to determine whether these antibodies contribute to disease pathology.

Hence, the presence of anti-galectin autoantibodies in the clinical situation in situ introduces a further level to be considered, their presence and significance warranting further efforts. In addition, anti-galectin autoantibodies may serve as a (prognostic) biomarker of disease progression. However, their presence may be a complicating factor when targeting galectin–glycan interactions to promote remyelination in MS lesions.

## Concluding remarks

Why remyelination ultimately fails in MS is still unknown. This review provides a survey of galectin involvement and reflects the multifunctionality of these tissue lectins. Indeed, galectins have multifaceted functions in modulating innate and adaptive immune responses in MS and are actively involved in regulating successful remyelination by affecting OPC differentiation in a direct and indirect manner. Galectins-1, -3 and -4 can be considered as factors that determine OPC differentiation and thereby (re)myelination by placing glial communication in the right time and spatial context. Identifying the biochemical nature of the counterreceptors and the chain of post-binding events as well as the antagonism/synergy between galectins in situ are open issues that need to be resolved. Also, a role of galectins that so far have not been investigated in the context of remyelination needs to be considered. For example, galectin-2, acting via the TLR4 pathway, skews macrophages toward a pro-inflammatory phenotype [318], which may also be valid in MS lesions. Interestingly, proto-type galectins-1, -2 and -7 induce apoptosis in activated T cells via different profiles of caspase activation [319]. In addition, involvement of members of other lectin families such as myelin-associated glycoprotein (siglec-4) could be worth exploring [320], and this on the network level considering various emerging modes of functional cooperation [321]. Altogether, the evidence and considerations presented in this review emphasize that dynamic expression, secretion and re-uptake of galectins, all in an intimately regulated manner, are crucial for remyelination to take place. Further research is definitely needed to understand the mechanisms that control the properties of galectins and their effect on OPC differentiation, astrocytes, microglia/macrophage activation, neurodegeneration and T cells. Thus, galectins do have potential as target or tool for the development of remyelination-based therapies in MS, but more work at the basic science level is required prior to attempting to realize their potential.
